# Novel exonic mutation inducing aberrant splicing in the *IL10RA* gene and resulting in infantile-onset inflammatory bowel disease: a case report

**DOI:** 10.1186/s12876-016-0424-5

**Published:** 2016-01-28

**Authors:** Tadahiro Yanagi, Tatsuki Mizuochi, Yugo Takaki, Keisuke Eda, Keiichi Mitsuyama, Masataka Ishimura, Hidetoshi Takada, Dror S. Shouval, Alexandra E. Griffith, Scott B. Snapper, Yushiro Yamashita, Ken Yamamoto

**Affiliations:** Department of Pediatrics and Child Health, Kurume University School of Medicine, 67 Asahi-machi, Kurume, 830-0011 Japan; Division of Gastroenterology Department of Medicine, Kurume University School of Medicine, Kurume, Japan; Department of Pediatrics, Graduate School of Medical Sciences, Kyushu University, Fukuoka, Japan; Division of Gastroenterology, Hepatology and Nutrition, Boston Children’s Hospital, Boston, MA USA; Harvard Medical School, Brigham and Women’s Hospital, Boston, MA USA; Division of Gastroenterology, Brigham and Women’s Hospital, Boston, MA USA; Department of Medical Chemistry, Kurume University School of Medicine, Kurume, Japan

**Keywords:** IL-10, IL-10 receptor, infantile-onset inflammatory bowel disease, hematopoietic stem cell transplantation

## Abstract

**Background:**

Although deleterious mutations in interleukin-10 and its receptor molecules cause severe infantile-onset inflammatory bowel disease, there are no reports of mutations affecting this signaling pathway in Japanese patients. Here we report a novel exonic mutation in the *IL10RA* gene that caused unique splicing aberrations in a Japanese patient with infantile-onset of inflammatory bowel disease in association with immune thrombocytopenic purpura and a transient clinical syndrome mimicking juvenile myelomonocytic leukemia.

**Case presentation:**

A Japanese boy, who was the first child of non-consanguineous healthy parents, developed bloody diarrhea, perianal fistula, and folliculitis in early infancy and was diagnosed with inflammatory bowel disease. He also developed immune thrombocytopenic purpura and transient features mimicking juvenile myelomonocytic leukemia. The patient failed to respond to various treatments, including elemental diet, salazosulfapyridine, metronidazole, corticosteroid, infliximab, and adalimumab. We identified a novel mutation (c.537G > A, p.T179T) in exon 4 of the *IL10RA* gene causing unique splicing aberrations and resulting in lack of signaling through the interleukin-10 receptor. At 21 months of age, the patient underwent allogeneic hematopoietic stem cell transplantation and achieved clinical remission.

**Conclusions:**

We describe a novel exonic mutation in the *IL10RA* gene resulting in infantile-onset inflammatory bowel disease. This mutation might also be involved in his early-onset hematologic disorders. Physicians should be familiar with the clinical phenotype of IL-10 signaling defects in order to enable prompt diagnosis at an early age and referral for allogeneic hematopoietic stem cell transplantation.

**Electronic supplementary material:**

The online version of this article (doi:10.1186/s12876-016-0424-5) contains supplementary material, which is available to authorized users.

## Background

Interleukin-10 (IL-10), an anti-inflammatory cytokine, binds to 2 receptors, namely 2 alpha molecules (IL-10RA/IL-10R1) and 2 beta molecules (IL-10RB/IL-10R2) [1, 2]. IL-10 signaling plays a key role in maintaining immune homeostasis in the gastrointestinal tract. Accordingly, defects of *IL10*, *IL10RA*, or *IL10RB* genes cause very early-onset inflammatory bowel disease (IBD) including infantile-onset IBD (IOIBD) [3–5]. Patients with mutations in *IL10* or IL10 receptor (*IL10R*) genes present with severe colitis, perianal disease and folliculitis manifesting in the first months of life. These patients are refractory to immunosuppressive therapies such as corticosteroids, methotrexate, thalidomide, and anti-tumor necrosis factor-alpha (TNF-α) antibodies, yet, allogeneic hematopoietic stem cell transplantation (HSCT) has been shown to be curative in these conditions [3–5]. To date over 40 patients of various ethnicities with *IL10*/*IL10R* deficiency have been reported [3–14]. However, reports on IL-10 signaling pathway defects in the Japanese population are lacking. Here we describe a Japanese IOIBD patient with a novel exonic mutation in the *IL10RA* gene causing unique splicing aberrations. Interestingly, this patient also developed immune thrombocytopenic purpura (ITP) and transient abnormalities mimicking juvenile myelomonocytic leukemia (JMML) in early infancy.

## Case presentation

A Japanese boy was born by spontaneous vaginal delivery at 41 weeks of gestation, with a birth weight of 2834 g. He was the first child of non-consanguineous healthy parents. At 2 months of age, he presented with hepatosplenomegaly, purpura, leukocytosis (white blood cell count, 2.5 × 10^10^ /L), monocytosis (monocytes, 2.0 × 10^9^ /L), anemia (hemoglobin, 87 g/L), and thrombocytopenia (platelets, 3.6 × 10^10^ /L). Bone marrow examination disclosed hypercellularity without excessive blasts or abnormal appearance of megakaryocytes. Cytogenetic analysis indicated a normal male karyotype, and colony assay of bone marrow mononuclear cells showed spontaneous colony formation and high sensitivity to granulocyte-macrophage colony stimulating factor. Genetic analysis revealed no reported mutations in *PTPN11*, *NRAS*, *KRAS*, or *CBL* genes. While his clinical manifestations met diagnostic criteria for JMML [15], his symptoms and blood test results normalized spontaneously.

At 6 months of age he developed bloody diarrhea, perianal fistula, and folliculitis. Endoscopy showed erosive changes, multiple aphthous ulcers, and longitudinal ulcerations of the colon. Elevated C-reactive protein and increased erythrocyte sedimentation rate suggested the presence of a chronic inflammatory disorder. Immune work-up was normal, including T and B lymphocytes numbers, proliferative response of peripheral blood mononuclear cells (PBMCs) to phytohemagglutinin, serum immunoglobulin concentrations, neutrophil phagocytic capacity and microbicidal activity. The Wiskott-Aldrich syndrome protein was expressed normally in PBMCs.

Based on clinical, laboratory, and endoscopic findings, the patient was diagnosed with Crohn’s disease, which was treated with an elemental diet, salazosulfapyridine, metronidazole, and a corticosteroid. At the same time, he again developed thrombocytopenia (platelets, 3.2 × 10^10^ /L) and was diagnosed with ITP since megakaryocytes in the bone marrow were normal and platelet-associated immunoglobulin G was elevated (90 ng/10^7^ cells) [16]. Treatment with high-dose intravenous immunoglobulin G led to prompt normalization of platelets count, but did not lead to any improvement in bloody diarrhea, fistula, or folliculitis. The patient also had recurrent fevers and was treated with systemic antibiotics.

Given lack of colitis improvement, at 9 months of age, he began treatment with infliximab (IFX), achieving partial response of bloody diarrhea, perianal fistula, and folliculitis. At the age of 14 months, adalimumab (ADA) was substituted for IFX because of an allergic reaction to infusions. From that point he was treated with a maintenance regimen of ADA, an elemental diet, and salazosulfapyridine. Growth and development then entered the normal range, although bloody diarrhea and folliculitis persisted. Given his clinical features, an IL-10 signaling defect was suspected and targeted sequencing confirmed an *IL10RA* mutation.

At 21 months of age, the patient underwent allogeneic HSCT using umbilical cord blood from a matched unrelated donor at Kyushu University Hospital, achieving rapid and sustained complete chimerism and clinical remission. Currently, at the age of 24 months, the patient is in sustained clinical remission since 2 months after HSCT.

### Identification of novel *IL10RA* mutation and IL-10 receptor defect

**Fig. 1:**
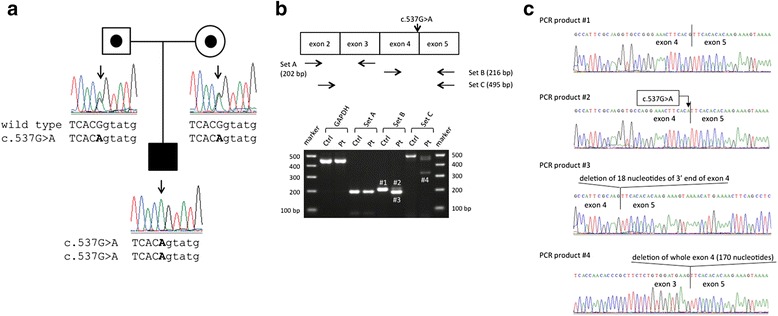
Identification of the *IL10RA* mutation associated with infantile-onset inflammatory bowel disease (IOIBD). **a** A c.537G > A mutation at the 3' end of exon 4 of the *IL10RA* gene in the family of our patient with IOIBD. Arrows indicate site of mutation. Parts of sequences of exon 4 and intron 4 are shown in upper case and lower case, respectively. **b** RT-PCR analysis for the *IL10RA* gene in the patient with c.537G > A mutation. Schematic representation of positions of the primers for RT-PCR and the c.537G > A mutation is given in the upper panel. RT-PCR products from the c.537G > A mutation of the *IL10RA* gene are shown in the lower panel. The expected 216-bp and 495-bp products of the *IL10RA* exons 4-5 and exons 2-5, respectively, were detected in a healthy control and the patient, while additional shorter products were observed in the patient (bands 3 and 4). **c** Sequence analysis of RT-PCR products indicated that the c.537G > A mutation caused 2 kinds of splicing variants; an 18 bp deletion of the 3' end of exon 4 and skipping of exon 4 (170 bp)

To detect mutations of *IL10RA* and *IL10RB*, we performed direct sequence analysis using genomic DNA isolated from PBMCs from the patient and his parents (see Additional files 1 and 2). We identified a novel homozygous and heterozygous mutation (c.537G > A, p.T179T) at the 3' end of exon 4 of *IL10RA* in the patient and his parents, respectively (Fig. 1a). Because the nucleotides at the end of the exon are involved in the splicing process, we examined whether the c.537G > A mutation generated splicing variants by reverse-transcription polymerase chain reaction (RT-PCR) analysis using 3 primer sets: set A, to amplify exons 2 and 3 as a control; set B, to detect deletion or insertion near the boundary between exons 4 and 5; and set C, to amplify exons 3 and 4 (upper panel in Fig. 1b). We detected an additional small PCR product in the patient, as well as the product of expected size using set B (compare band #3 with #2, lower panel in Fig. 1b). Moreover, we found a much smaller product in the patient using set C (band #4, lower panel in Fig. 1b). These results suggested that the exonic c.537G > A mutation generated at least 2 abnormal transcripts of the *IL10RA* gene. We further investigated the nucleotide sequence of these PCR products. As shown in Fig. 1c, product #2, which showed expected size, possessed a c.537G > A mutation but indicated intact splicing between exon 4 and 5. However, product #3 showed an 18-base deletion at the 3' end of exon 4, which was generated by activation of a nearby cryptic splice donor site. This 18-base deletion was predicted to cause loss of 6 amino acids (174-VPGNFT-179) from the IL-10RA molecule. Moreover, product #4 showed complete deletion of exon 4 (170 bases). This exon 4 skipping caused a frameshift, with translational arrest at a site 7 amino acids distant. Thus, the exonic c.537G > A mutation induced aberrant splicing in the *IL10RA* gene, which would cause loss of function.

**Fig. 2:**
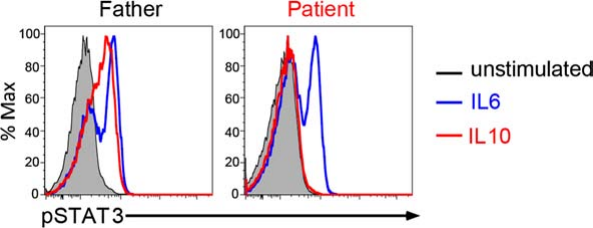
Loss of function of the IL-10 receptor. Functional analysis of IL-10 receptor complex was performed by determining signal transducer and activator of transcription 3 (STAT3) phosphorylation using flow cytometry. Peripheral blood mononuclear cells from the patient (right panel) and his father (left panel) were stimulated for 15 minutes with IL-10 (20 ng/mL) or IL-6 (20 ng/mL) or kept unstimulated, and later fixed, permeabilized and stained for phosphorylated STAT3 (pSTAT3), which is downstream of the IL-10 and IL-6 receptor complexes

To determine whether the novel mutation identified in the patient resulted in defective signaling function involving the IL-10R, PBMCs obtained from the patient were compared with those from his father, who served as a healthy control. Cells were stimulated with IL-10 as previously described [13] (see Additional file 3). IL-10 stimulation of the patient’s PBMCs failed to induce phosphorylation of signal transducer and activator of transcription 3 (pSTAT3), which is a key transcription factor downstream of the IL-10R. IL-6-induced pSTAT3, which served as an internal positive control, was intact (right panel in Fig. 2). In contrast, both IL-10 stimulation and IL-6 stimulation of the control cells led to pSTAT3 formation (left panel in Fig. 2). These results suggest that the mutation resulted in loss of normal function of the IL-10R.

## Discussion

Loss of function of IL-10 and IL-10R molecules causes refractory colitis with folliculitis and perianal disease, manifesting very early in infancy [3–5]. Here we report a Japanese IOIBD patient with a novel exonic mutation (c.537G > A, p.T179T) in exon 4 of the *IL10RA* gene that produced uniquely abnormal transcripts. Also in early infancy, the patient developed ITP and a transient clinical syndrome with features mimicking JMML.

From early infancy, the patient manifested refractory colitis, perianal disease and folliculitis, in addition to associated hematologic disorders, which may also be the result of the *IL10RA* genetic defect. Expression of IL-10 in PBMCs has been reported to correlate to some extent with disease activity in children with chronic active ITP [17]. IL-10 normally exerts negative feedback control that limits activation of Th1 cells as well as monocytes and macrophages [18]. In vitro, IL-10 has been found to inhibit cell growth and granulocyte/macrophage colony stimulating factor production in chronic myelomonocytic leukemia cells, as well as inhibiting cytokine production and growth in JMML cells [19, 20]. Neven et al and Shouval et al reported that patients with IL-10R deficiency tend to develop non-Hodgkin B-cell lymphomas [13, 21]. These findings suggest that IL-10 signaling defects might lead not only to development of lymphoma but also to ITP and JMML-like states. However, there is a possibility that there may be another gene involved here as the remainder of this patient’s genes have not been tested.

We identified a point mutation, c.537G > A, in the exon adjacent to a splice donor site whose consensus sequence is MAG|GURAGU (M represents A or C, while R represents a purine), extending from position -3 to position +6 relative to the exon–intron junction. Within the consensus sequence, mutations affecting GU residues at positions +1 and +2 are most common followed by mutations at position +5 [22]. In our patient, a relatively rare -1G > -1A homozygous mutation resulted in aberrant splicing manifest as disease. The AG|GURAGU motif is recognized by the U1 small nuclear ribonucleoprotein (snRNP) to define the exon-intron boundary in the first step of splicing process [23]. When the motif is changed, U1 snRNP would not recognize the exon-intron junction properly and would bind to other regions with similar sequence. In the exon 4 of *IL10RA*, there is AGGTGCCA sequence at 18 bases upstream of exon 4-intron 4 boundary where c.537G > A is located. This sequence might be recognized by U1 snRNP, and the splicing variant of 18-base deletion at the 3' end of exon 4 was generated in our patient. However, the AGGTGCCA sequence in exon 4 is not completely matched to the conserved AGGURAGU motif, so that another splicing variant, exon 4 skipping, might occur alternatively. This type of splicing aberration is not specific to the case in this study because we obtained similar results in the patients affected with familial hemophagocytic lymphohistiocytosis previously [24, 25].

According to the database which predicts splice-altering single nucleotide variants (dbscSNV: https://www.solvebio.com/library/dbscSNV), the *IL10RA* c.537G > A substitution shows high probability of affecting splicing (the Ada score = 0.99996). This *in silico* analysis strongly supports our results that the c.537G > A mutation generated splicing variants. Although the c.537G > A nucleotide change is not found in the three databases (1000 Genome: http://www.1000genomes.org/, ExAC: http://exac.broadinstitute.org/, or NHLBI Exome Variant Server: http://evs.gs.washington.edu/EVS/), the frequency of this variant is shown to be 0.0019 in the Human Genetic Variation Database which contains genetic variations determined by exome sequencing of 1208 Japanese (http://www.genome.med.kyoto-u.ac.jp/SnpDB/). This suggests that the c.537G > A mutation would be detected in other Japanese IOIBD patients in future.

The c.537G > A mutation caused an in-frame deletion of 18 bp of exon 4 in the *IL10RA* gene that leads to loss of 6 amino acids (174-VPGNFT-179). Structural analysis of the IL-10/IL-10RA/IL-10RB complex suggests that these amino acids compose a hydrophobic area that could importantly influence crucial interactions with IL-10RB molecules [26]. Because sequential assembly of IL10-RA and IL-10RB is required for IL-10 cellular response [27], disruption of stable interactions between these molecules could lead to a loss of IL-10 signaling, resulting in IOIBD in our patient. Although we also identified the normal splicing PCR product in our patient, we speculated that the normal splicing produces a small amount of IL-10RA and cannot rescue the IL-10 signaling in his macrophages and T cells.

## Conclusions

We identified a novel exonic mutation (c.537G > A, p.T179T) in the *IL10RA* gene that caused unique splicing aberrations likely to lead to IOIBD. IL-10R genetic defects also might be involved in hematologic disorders during infancy such as ITP and JMML-like disorders. Physicians should be familiar with the clinical phenotype of IL-10 signaling defects in order to enable prompt diagnosis at an early age and referral for allogeneic HSCT, before later complication occur, such as development of lymphoma.

## Consent

Written informed consent was obtained from the patient’s parents for publication of this case report and any accompanying images. A copy of the written consent is available for review by the Editor of this journal. This study was approved by the Institutional Review Board at Kurume University, Japan and the Boston Children’s Hospital, MA.

## Additional files

Additional file 1:
**Genetic analysis of the** ***IL10RA*** **and** ***IL10RB*** **genes.** (DOCX 13 kb) Additional file 2:
**Primer sets for mutation screening of** ***IL10RA*** **and** ***IL10RB*** **genes.** (DOCX 15 kb) Additional file 3:
**Functional assay of the IL-10 receptor.** (DOCX 14 kb)

## Abbreviations

IL-10: interleukin-10
IL-10RA: IL-10 receptor alpha subunit
IL-10RB: IL-10 receptor beta subunit
IBD: inflammatory bowel disease
IL-10R: interleukin-10 receptor
IOIBD: infantile-onset inflammatory bowel disease
TNF-α: tumor necrosis factor-alpha
HSCT: hematopoietic stem cell transplantation
ITP: immune thrombocytopenic purpura
JMML: juvenile myelomonocytic leukemia
PBMCs: peripheral blood mononuclear cells
IFX: infliximab
ADA: adalimumab
RT-PCR: reverse-transcription polymerase chain reaction
pSTAT3: phosphorylation of signal transducer and activator of transcription 3
snRNP: small nuclear ribonucleoprotein
