# Supplementary material for: Novel exonic mutation inducing aberrant splicing in the IL10RA gene and resulting in infantile-onset inflammatory bowel disease: a case report
Source: BMC Gastroenterol. 2016 Jan 28;16:10. doi: 10.1186/s12876-016-0424-5 (PMC4730728; doi:10.1186/s12876-016-0424-5)
Supplement: Additional file 1: — Genetic analysis of the IL10RA and IL10RB genes. (DOCX 13 kb) [file 12876_2016_424_MOESM1_ESM.docx]

**Additional file 1**

**Genetic analysis of the *IL10RA* and *IL10RB* genes**

For the detection of *IL10RA* or *IL10RB* mutations, genomic DNA was isolated from peripheral blood cells from the patient and his parents. Genomic DNA (20 ng) was subjected to PCR using the primers listed in Additional file 2. These primer sets were designed to amplify 7 exons including the coding regions with the exon-intron boundaries of the *IL10RA* and *IL10RB* genes. The PCR products were treated with ExoSAP-IT (Affymetrix, Santa Clara, CA) by incubation at 37ºC for 15 minutes to inactivate the free primers and dNTPs, and then subjected to sequencing reactions using forward or reverse primers and BigDye Terminator v3.1 (Life Technologies, Carlsbad, CA). The DNA fragments were purified using a Centri-Sep spin column (Life Technologies), and sequencing was carried out with an ABI 3100 Genetic Analyzer (Life Technologies). Sample sequences were aligned to reference sequences obtained from the UCSC Genome Bioinformatics website (http://genome.ucsc.edu/index.html) using the ClustalW program in order to identify nucleotide changes. Mutations were numbered according to GenBank Reference Sequence NM_001558.3 (*IL10RA*); additionally, the A of the ATG initiator codon was defined as nucleotide +1.

To identify splicing variants generated by c.537G>A mutation of the *IL10RA* gene, total RNA was extracted from peripheral blood cells and reverse-transcription PCR (RT-PCR) was performed. Primers for RT-PCR were as follows: set A, 5'-TCCGTCTGTGTGGTTTGAAG-3' and 5'-GTAGCCATTGCTGTGGTACA-3'; set B, 5'-TCCTCGGGAAGATTCAGCTA-3' and 5'-AAGCGACAGATGGTTTCACC-3'; and set C, 5'-CACCCATCCCAAATCAGTCT-3' and 5'-AAGCGACAGATGGTTTCACC-3'; GAPDH, 5'-ACCACAGTCCATGCCATCAC-3' and 5'-TCCACCACCCTGTTGCTGTA-3'. The PCR products were cloned into a TA cloning vector (Life Technologies), and the sequences were verified with an ABI 3100 DNA Genetic Analyzer.
