# Supplementary material for: Novel exonic mutation inducing aberrant splicing in the IL10RA gene and resulting in infantile-onset inflammatory bowel disease: a case report
Source: BMC Gastroenterol. 2016 Jan 28;16:10. doi: 10.1186/s12876-016-0424-5 (PMC4730728; doi:10.1186/s12876-016-0424-5)
Supplement: Additional file 2: — Primer sets for mutation screening of IL10RA and IL10RB genes. (DOCX 15 kb) [file 12876_2016_424_MOESM2_ESM.docx]

**Additional file 2**

**Primer sets for mutation screening of *IL10RA* and *IL10RB* genes**

| Primer name | Forward primer sequence | Reverse primer sequence | Target exon | Expected size (bp) | Primer for sequencing |
| --- | --- | --- | --- | --- | --- |
| *IL10RA* |  |  |  |  |  |
| IL10RA-1 | AGTCCCAGCCCAAGGGTAG | TCTCCACTGGATGGAGAACTTT | 1 | 219 | Forward |
| IL10RA-2 | GCCCACTCTGCCCCTTAC | CTCCCTCTTCCCTTTTCCTC | 2 | 282 | Forward |
| IL10RA-3 | CCTCTTGCGTCTCCCTTAAA | GGGACTTCAGAGCCATGTTC | 3 | 296 | Forward |
| IL10RA-4 | CTGCATTGACAAACCTGTGG | GAAGCCAAGGGAGTTCTGG | 4 | 357 | Forward |
| IL10RA-5 | TAAAGGCCCACCAGCTCTC | GAGAGCAGCCCAGCTGAC | 5 | 250 | Forward |
| IL10RA-6 | GTGGCCTGTAGTTTGCCTTG | CAGTGACCTCCTTGCAAGACT | 6 | 285 | Forward |
| IL10RA-7-1 | CTCCATCGAGCTCTCCTCCT | CCCTGTTTCCCAGCGTTC | 7 | 345 | Forward |
| IL10RA-7-2 | CACGGCAGCACAGACAGT | ACAGCAGCTGGGTCTTCTTC | 7 | 383 | Forward |
| IL10RA-7-3 | CAGGATGACAGTGGCATTGA | ATTCCTCAGTGGGCTGGTTC | 7 | 376 | Forward |
| IL10RA-7-4 | CAGGCTTGCATCCACCAG | GAGCAGGCATGGCTAAAATC | 7 | 326 | Forward |
| *IL10RB* |  |  |  |  |  |
| IL10RB-1 | CGTGTGCTTGGAGGAAGC | GTGGCGTTTGCATCTTCTCT | 1 | 226 | Reverse |
| IL10RB-2 | GTGGCCTTTGAAGACATGGA | GCGTTCCTGCCAATAGTGAG | 2 | 269 | Forward |
| IL10RB-3 | ATGTTTAACACAGTTTCCACTCC | TTGATGGACCAACCAATGTG | 3 | 351 | Forward |
| IL10RB-4 | TCATGACAATAGTATATCAAAGCAGTG | GGACCAGTCCATAAGGTGCT | 4 | 383 | Forward |
| IL10RB-5 | TGCCCTTCCACTGCTTAGTC | CTGAGCTGGAATTTGAGTTGG | 5 | 290 | Reverse |
| IL10RB-6 | CCCCCAAGATAAACGTACAGG | TCCACATCTCACTCCATCCT | 6 | 299 | Forward |
| IL10RB-7 | AACTGTCCTCTTTCCCTCCA | AGATGGTCTTGGCCCTTGTT | 7 | 298 | Reverse |
