# Supplementary material for: Novel exonic mutation inducing aberrant splicing in the IL10RA gene and resulting in infantile-onset inflammatory bowel disease: a case report
Source: BMC Gastroenterol. 2016 Jan 28;16:10. doi: 10.1186/s12876-016-0424-5 (PMC4730728; doi:10.1186/s12876-016-0424-5)
Supplement: Additional file 3: — Functional assay of the IL-10 receptor. (DOCX 14 kb) [file 12876_2016_424_MOESM3_ESM.docx]

**Additional file 3**

**Functional assay of the IL-10 receptor**

Blood samples obtained from the patient and his father, who severed as a healthy control, were sent overnight to Dr. Snapper’s laboratory in Boston, MA. PBMCs were isolated by a Ficoll-Paque PLUS (GE Healthcare, Boston MA) gradient, according to manufacturer’s instructions. Cells were stimulated with IL-10 or IL-6 (both 20 ng/mL) for 15 minutes or kept unstimulated, and then fixed, permeabilized, and stained for pSTAT3 (all from BD Biosciences, San Jose, CA). Signaling through the IL-10 receptor was determined by degree of phosphorylation of STAT3 following IL-10 stimulation, compared to the unstimulated condition. IL-6 stimulation served as a positive internal control, since it also leads to phosphorylation of STAT3.
